# Supplementary material for: Movement and joints: effects of overuse on anuran knee tissues
Source: PeerJ. 2018 Aug 31;6:e5546. doi: 10.7717/peerj.5546 (PMC6120441; doi:10.7717/peerj.5546)
Supplement: Supplemental Information 1 — MCV: field number of Miriam Corina Vera. [file peerj-06-5546-s001.doc]

**Appendix**

List of treated and control specimens. MCV: field number of Miriam Corina Vera.

*Leptodactylus latinasus,* juveniles:MCV 108, 109, 110, 262, 305, 382; adults: MCV 409 (female), MCV 450 (male), MCV 451(male), MCV 452 (male), and MCV 453 (male). *Leptodactylus mystacinus* (juveniles)*:* MCV 42, 49, 50, 52, 57, 82, 97, 98, 99 and 100; adults: MCV 532-541(males). *Melanophryniscus rubriventris,* adults: MCV 128 (female), MCV 434 (female), MCV 436 (male), MCV 437 (male), MCV 438 (male), MCV 439 (male), 440 (female). *Phyllomedusa sauvagii,* juveniles: MCV 67, 76, 81; adults: MCV 78 (female), 88 (male), 107 (male), 443 (female) and MCV 444 (female). *Rhinella arenarum,* juveniles: MCV 104, 105,445, 446, 447, 455 and 456; adults: MCV 520 (male), 521 (male), 522 (female), 523 (male), 524 (female), 525-531 (males).
