# Supplementary material for: Movement and joints: effects of overuse on anuran knee tissues
Source: PeerJ. 2018 Aug 31;6:e5546. doi: 10.7717/peerj.5546 (PMC6120441; doi:10.7717/peerj.5546)
Supplement: Supplemental Information 2 — mNFm: nuclei of the fibrocartilage; mFTM: tendon fibers; mFMm: menisci fibers; mCHm, hypertrophic chondrocytes; st: stage; sp: specie; tr, treatment; md: locomotor mode; int1: intercept sp*tr; int2: intercept st*tr; int3: intercept md*tr. [file peerj-06-5546-s002.docx]

Results of multinomial ordinal logistic regression analysis. mNFm: nuclei of the fibrocartilage; mFTM: tendon fibers; mFMm: menisci fibers; mCHm, hypertrophic chondrocytes; st: stage; sp: specie; tr, treatment; md: locomotor mode; int1: intercept sp*tr; int2: intercept st*tr; int3: intercept md*tr.

| **Collagen fibers of the fibrocartilage models** |  |  | **Value** | **Std. error** | **T value** |
| --- | --- | --- | --- | --- | --- |
| 1) mFFm | Intercepts | 0\|1 | 0.1292 | 0.2545 | 0.5077 |
|  |  | 1\|2 | 0.1292 | 0.4664 | 5.2177 |
|  |  |  |  |  |  |
| 2) mFFm.tr | Coefficients | Treated | 0.7446 | 0.5039 | 1.478 |
|  |  |  |  |  |  |
|  | Intercepts | 0\|1 | 0.4971 | 0.3629 | 1.3696 |
|  |  | 1\|2 | 2.8547 | 0.5586 | 5.1108 |
|  |  |  |  |  |  |
| 3) mFFm.sp.tr | Coefficients | *L. mystacinus* | -1.070 | 0.8006 | -1.337 |
|  |  | *M. rubriventris* | 0.0292 | 0.9467 | 0.0306 |
|  |  | *P. sauvagii* | -0.40399 | 0.9305 | -0.4316 |
|  |  | *R. arenarum* | 0.48996 | 0.7443 | 0.61684 |
|  |  | Treated | 0.81191 | 0.5316 | 1.52740 |
|  |  |  |  |  |  |
|  | Intercepts | 0\|1 | 0.2591 | 0.6548 | 0.3956 |
|  |  | 1\|2 | 2.7859 | 0.7912 | 3.5212 |
|  |  |  |  |  |  |
| 4) mFFm.md.tr | Coefficients | Jumper | -0.06769 | 0.5674 | -0.1193 |
|  |  | Treated | 0.74177 | 0.5045 | 1.4704 |
|  |  |  |  |  |  |
|  | Intercepts | 0\|1 | 0.4459 | 0.5614 | 0.7943 |
|  |  | 1\|2 | 2.8033 | 0.7038 | 3.9831 |
|  |  |  |  |  |  |
| 5) mFFm.st.tr | Coefficients | Juvenile | -1.0935 | 0.5446 | -2.008 |
|  |  | Treated | 0.8228 | 0.5196 | 1.583 |
|  |  |  |  |  |  |
|  | Intercepts | 0\|1 | 0.1084 | 0.4105 | 0.2640 |
|  |  | 1\|2 | 2.5719 | 0.5779 | 4.4506 |
|  |  |  |  |  |  |
| 6) mFFm.sp.st.tr | Coefficients | *L. mystacinus* | -1.2471 | 0.8134 | -1.5333 |
|  |  | *M. rubriventris* | -0.6204 | 1.0043 | -0.6178 |
|  |  | *P. sauvagii* | -0.6935 | 0.9703 | -0.7147 |
|  |  | *R. arenarum* | 0.2199 | 0.7975 | 0.2758 |
|  |  | Juvenile | -1.1296 | 0.5970 | -1.5921 |
|  |  | Treated | 0.8882 | 0.5447 | 1.6307 |
|  |  |  |  |  |  |
|  | Intercepts | 0\|1 | -0.3776 | 0.7301 | -0.5171 |
|  |  | 1\|2 | 2.2823 | 0.8202 | 2.7826 |
|  |  |  |  |  |  |
| 7) mFFm.md.st.tr | Coefficients | Jumper | 0.2423 | 0.6050 | 0.4004 |
|  |  | Juvenile | -1.1496 | 0.5643 | -2.0374 |
|  |  | Treated | 0.8374 | 0.5216 | 1.6054 |
|  |  |  |  |  |  |
|  | Intercepts | 0\|1 | 0.2735 | 0.5837 | 0.4687 |
|  |  | 1\|2 | 2.7470 | 0.7306 | 3.7601 |
|  |  |  |  |  |  |
| 8) mFFm.int1 | Coefficients | *L. mystacinus* | 0.2725 | 1.066 | 2.557e-01 |
|  |  | *M. rubriventris* | 1.4854 | 1.282 | 1.159e+00 |
|  |  | *P. sauvagii* | -0.0000115 | 1.484 | -7.749e-06 |
|  |  | *R. arenarum* | -0.2148694 | 1.94 | -1.800e-01 |
|  |  | Treated | 2.2510772 | 1.399 | 1.609e+00 |
|  |  | *L. mystacinus:*treated | -3.1974613 | 1.730 | -1.848e+00 |
|  |  | *M. rubriventris:*treated | -3.7364416 | 2.100 | -1.780e+00 |
|  |  | *P. sauvagii:*treated | -1.2619762 | 1.029 | -6.221e-01 |
|  |  | *R. arenarum:*treated | 0.985238 | 1.743 | 5.625e-01 |
|  |  |  |  |  |  |
|  | Intercepts | 0\|1 | 0.7270 | 0.8571 | 0.8481 |
|  |  | 1\|2 | 3.7752 | 1.0564 | 3.5736 |
|  |  |  |  |  |  |
| 9) mFFm.int2 | Coefficients | Juvenile | -0.9966 | 0.7978 | -1.2492 |
|  |  | Treated | 0.8852 | 0.6446 | 1.3733 |
|  |  | Juvenile: treated | -0.1779 | 1.0816 | -0.1645 |
|  |  |  |  |  |  |
|  | Intercepts | 0\|1 | 0.1376 | 0.4482 | 0.3071 |
|  |  | 1\|2 | 2.6066 | 0.6172 | 4.2233 |
|  |  |  |  |  |  |
| 10) mFFm.int3 | Coefficients | Jumper | -0.7756 | 0.8171 | -0.9492 |
|  |  | Treated | -0.2263 | 0.9615 | -0.2353 |
|  |  | Jumper: treated | 1.3330 | 1.1340 | 1.1755 |
|  |  |  |  |  |  |
|  | Intercepts | 0\|1 | -0.0794 | 0.6981 | -0.1137 |
|  |  | 1\|2 | 2.3111 | 0.7984 | 2.8948 |
| **Fibrocartilage Nuclei**  **models** |  |  | **Value** | **Std. error** | **T value** |
| 1) mNFm | Intercepts | 0\|1 | 0.1671 | 0.2897 | 0.5767 |
|  |  | 1\|2 | 2.1518 | 0.4725 | 4.5540 |
|  |  |  |  |  |  |
| 2) mNFm.tr | Coefficients | Treated | 0.9443 | 0.5765 | 1.638 |
|  |  |  |  |  |  |
|  | Intercepts | 0\|1 | 0.6150 | 0.4102 | 1.4994 |
|  |  | 1\|2 | 2.6789 | 0.5905 | 4.5367 |
|  |  |  |  |  |  |
| 3) mmNFm.sp.tr | Coefficients | *L. mystacinus* | -2.7538 | -2.7538 | -2.989e+00 |
|  |  | *M. rubriventris* | 0.1279 | 8.865e-01 | 1.443e-01 |
|  |  | *P. sauvagii* | 0.3086 | 9.361e-01 | 3.296e-01 |
|  |  | *R. arenarum* | -18.8101 | 4.109e-08 | -4.577e+08 |
|  |  | Treated | 1.4031 | 1.4031 | 1.979e+00 |
|  |  |  |  |  |  |
|  | Intercepts | 0\|1 | -2.29100e-01 | 6.470000e-01 | -3.54100e-01 |
|  |  | 1\|2 | 2.49290e+00 | 8.14000e-01 | 3.062500e+00 |
|  |  |  |  |  |  |
| 4) mNFm.md.tr | Coefficients | Jumper | -1.653 | 0.6425 | -2.572 |
|  |  | Treated | 1.006 | 0.6708 | 1.665 |
|  |  |  |  |  |  |
|  | Intercepts | 0\|1 | -0.4757 | 0.5967 | -0.7972 |
|  |  | 1\|2 | 1.8704 | 0.6708 | 2.7881 |
|  |  |  |  |  |  |
| 5) mNFm.st.tr | Coefficients | Juvenile | -0.2501 | 0.5794 | -0.4317 |
|  |  | Treated | 0.9556 | 0.5781 | 1.6530 |
|  |  |  |  |  |  |
|  | Intercepts | 0\|1 | 0.5122 | 0.4720 | 1.0851 |
|  |  | 1\|2 | 2.5836 | 0.6281 | 4.1133 |
|  |  |  |  |  |  |
| 6) mNFm.sp.st.tr | Coefficients | *L. mystacinus* | -2.7880 | 9.374e-01 | -2.974e+00 |
|  |  | *M. rubriventris* | 0.4942 | 9.904e-01 | 4.990e-01 |
|  |  | *P. sauvagii* | 0.4090 | 9.411e-01 | 4.346e-01 |
|  |  | *R. arenarum* | -19.2064 | 3.367e-08 | -5.705e+08 |
|  |  | Juvenile | 0.6647 | 7.619e-01 | 8.724e-01 |
|  |  | Treated | 1.3877 | 7.112e-01 | 1.951e+00 |
|  |  |  |  |  |  |
|  | Intercepts | 0\|1 | 1.175000e-01 | 7.664000e-01 | 1.533000e-01 |
|  |  | 1\|2 | 2.873100e+00 | 9.381000e-01 | 3.062700e+00 |
|  |  |  |  |  |  |
| 7) mNFm.md.st.tr | Coefficients | Jumper | -1.8626 | 0.7111 | -2.6193 |
|  |  | Juvenile | 0.4788 | 0.6616 | 0.7237 |
|  |  | Treated | 0.9835 | 0.6052 | 1.6252 |
|  |  |  |  |  |  |
|  | Intercepts | 0\|1 | -0.4101 | 1.9605 | -0.6786 |
|  |  | 1\|2 | 1.9605 | 0.6857 | 2.8591 |
|  |  |  |  |  |  |
| 8) mFFm.int1 | Coefficients | *L. mystacinus* | -2.67265 | 1.307e+00 | -2.045e+00 |
|  |  | *M. rubriventris* | 0.26666 | 1.210e+00 | 2.204e-01 |
|  |  | *P. sauvagii* | -1.19929 | 1.435e+00 | -8.355e-01 |
|  |  | *R. arenarum* | -18.25922 | 4.287e-08 | -4.259e+08 |
|  |  | Treated | 1.00533 | 1.210e+00 | 8.310e-01 |
|  |  | *L. mystacinus:*treated | -0.07318 | 1.791e+00 | -4.086e-02 |
|  |  | *M. rubriventris:*treated | -0.26675 | 1.841e+00 | -1.449e-01 |
|  |  | *P. sauvagii:*treated | 2.76486 | 1.986e+00 | 1.392e+00 |
|  |  | *R. arenarum:*treated | -0.69315 | 1.126e-07 | -6.154e+06 |
|  |  |  |  |  |  |
|  | Intercepts | 0\|1 | -4.69000e-01 | 7.743000e-01 | -6.05700e-01 |
|  |  | 1\|2 | 2.479600e+00 | 9.572000e-01 | -6.05700e-01 |
|  |  |  |  |  |  |
| 9) mFFm.int2 | Coefficients | Juvenile | 0.1598 | 0.8245 | 0.1938 |
|  |  | Treated | 1.3017 | 0.7725 | 1.6850 |
|  |  | Juvenile: treated | -0.7991 | 1.1578 | -0.6902 |
|  |  |  |  |  |  |
|  | Intercepts | 0\|1 | 0.6806 | 0.5436 | 1.2520 |
|  |  | 1\|2 | 2.7719 | 0.6968 | 3.9783 |
|  |  |  |  |  |  |
| 10) mNFm.int3 | Coefficients | Jumper | -1.015 | 0.8758 | -1.158 |
|  |  | Treated | 1.868 | 1.0366 | 1.802 |
|  |  | Jumper: treated | -1.330 | 1.2672 | 1.802 |
|  |  |  |  |  |  |
|  | Intercepts | 0\|1 | -0.0929 | 0.7033 | -0.1320 |
|  |  | 1\|2 | 2.3841 | 0.8626 | 2.7637 |
| **Collagen fibers of the tendon models** |  |  | **Value** | **Std. error** | **T value** |
| 1) mFTm | Intercepts | 0\|1 | 0.4187 | 0.2575 | 1.6259 |
|  |  | 1\|2 | 2.9957 | 0.5916 | 5.0637 |
|  |  |  |  |  |  |
| 2) mFFm.tr | Coefficients | Treated | 0.5781 | 0.5132 | 1.126 |
|  |  |  |  |  |  |
|  | Intercepts | 0\|1 | 0.7009 | 0.3671 | 1.9092 |
|  |  | 1\|2 | 3.3069 | 0.6615 | 4.9995 |
|  |  |  |  |  |  |
| 3) mFTm.sp.tr | Coefficients | *L. mystacinus* | -0.8242 | 1.006e+00 | -8.191e-01 |
|  |  | *M. rubriventris* | 0.1395 | 1.191e+00 | 1.171e-01 |
|  |  | *P. sauvagii* | -18.8572 | 5.093e-08 | -3.702e+08 |
|  |  | *R. arenarum* | 19.7074 | 2.433e-03 | 8.099e+03 |
|  |  | Treated | 2.0750 | 8.614e-01 | 2.409e+00 |
|  |  |  |  |  |  |
|  | Intercepts | 0\|1 | 2.167900e+00 | 9.549000e-01 | 9.573000e-01 |
|  |  | 1\|2 | 2.256440e+01 | 9.573000e-01 | 2.357170e+01 |
|  |  |  |  |  |  |
| 4) mFTm.md.tr | Coefficients | Jumper | 2.1443 | 0.8225 | 2.607 |
|  |  | Treated | 0.7699 | 0.5522 | 1.394 |
|  |  |  |  |  |  |
|  | Intercepts | 0\|1 | 2.4873 | 0.8430 | 2.9505 |
|  |  | 1\|2 | 5.2745 | 1.0566 | 4.9921 |
|  |  |  |  |  |  |
| 5) mFTm.st.tr | Coefficients | Juvenile | -0.5156 | 0.5293 | -0.9741 |
|  |  | Treated | 0.6178 | 0.5186 | 1.1912 |
|  |  |  |  |  |  |
|  | Intercepts | 0\|1 | 0.5058 | 0.4145 | 1.2201 |
|  |  | 1\|2 | 3.1326 | 0.6814 | 4.5973 |
|  |  |  |  |  |  |
| 6) mFTm.sp.st.tr | Coefficients | *L. mystacinus* | -0.9120 | 1.047e+00 | -8.714e-01 |
|  |  | *M. rubriventris* | -0.5152 | 1.288e+00 | -4.000e-01 |
|  |  | *P. sauvagii* | -20.2186 | 2.881e-08 | -7.018e+08 |
|  |  | *R. arenarum* | 20.7144 | 2.257e-03 | 9.177e+03 |
|  |  | Juvenile | -1.2469 | 8.684e-01 | -1.436e+00 |
|  |  | Treated | -1.2469 | 8.902e-01 | 2.526e+00 |
|  |  |  |  |  |  |
|  | Intercepts | 0\|1 | 1.627000e+00 | 1.013200e+00 | 1.605800e+00 |
|  |  | 1\|2 | 2.321680e+01 | 9.744000e-01 | 2.382560e+01 |
|  |  |  |  |  |  |
| 7) mFTm.md.st.tr | Coefficients | Jumper | 2.5387 | 0.8718 | 2.912 |
|  |  | Juvenile | -1.0887 | 0.5952 | -1.829 |
|  |  | Treated | -1.0887 | 0.5755 | 1.597 |
|  |  |  |  |  |  |
|  | Intercepts | 0\|1 | 2.4087 | 0.8604 | 2.7996 |
|  |  | 1\|2 | 5.3172 | 1.0868 | 4.8926 |
|  |  |  |  |  |  |
| 8) mFTm.int1 | Coefficients | *L. mystacinus* | 2.035e+01 | 1.970e+01 | 1.033e+00 |
|  |  | *M. rubriventris* | 2.133e+01 | 1.971e+01 | 1.082e+00 |
|  |  | *P. sauvagii* | -1.143e-06 | 2.257e-04 | -5.063e-03 |
|  |  | *R. arenarum* | 3.958e+01 | 2.191e+00 | 1.807e+01 |
|  |  | Treated | 2.283e+01 | 1.969e+01 | 1.160e+00 |
|  |  | *L. mystacinus:*treated | -2.201e+01 | 1.971e+01 | -1.116e+00 |
|  |  | *M. rubriventris:*treated | -2.243e+01 | 1.973e+01 | -1.137e+00 |
|  |  | *P. sauvagii:*treated | -2.097e+01 | 3.491e-07 | -6.007e+07 |
|  |  | *R. arenarum:*treated | -1.134e+01 | 1.159e+02 | -9.789e-02 |
|  |  |  |  |  |  |
|  | Intercepts | 0\|1 | 2.242950e+01 | 1.969100e+01 | 1.139100e+00 |
|  |  | 1\|2 | 5.158280e+01 | 1.333654e+02 | 3.868000e-01 |
|  |  |  |  |  |  |
| 9) mFTm.int2 | Coefficients | Juvenile | -0.8321 | 0.7952 | -1.0465 |
|  |  | Treated | 0.3897 | 0.6628 | 0.5879 |
|  |  | Juvenile: treated | 0.5881 | 1.0737 | 0.5477 |
|  |  |  |  |  |  |
|  | Intercepts | 0\|1 | 0.3989 | 0.4528 | 0.8810 |
|  |  | 1\|2 | 3.0260 | 0.7038 | 4.2997 |
|  |  |  |  |  |  |
| 10) mFTm.int3 | Coefficients | Jumper | 1.5522 | 1.35918 | 1.142 |
|  |  | Treated | -0.1322 | -0.08787 | 1.504 |
|  |  | Jumper: treated | 1.0346 | 0.63997 | 1.617 |
|  |  |  |  |  |  |
|  | Intercepts | 0\|1 | 1.9556 | 1.0678 | 1.8314 |
|  |  | 1\|2 | 4.7604 | 1.2241 | 3.8891 |
| **Collagen fiber of the menisci models:** |  |  | **Value** | **Std. error** | **T value** |
|  |  |  |  |  |  |
| 1) mFMm | Intercepts | 0\|1 | -1.0586 | 0.2815 | -3.7608 |
|  |  | 1\|2 | -0.3677 | 0.2504 | -1.4688 |
|  |  |  |  |  |  |
| 2) mFMm.tr | Coefficients | Treated | -0.03944 | 0.4852 | -0.08129 |
|  |  |  |  |  |  |
|  | Intercepts | 0\|1 | -1.0788 | 0.3769 | -2.8621 |
|  |  | 1\|2 | -0.3880 | 0.3537 | -1.0969 |
|  |  |  |  |  |  |
| 3) mFMm.sp.tr | Coefficients | *L. mystacinus* | -1.28136 | 7.578e-01 | -1.691e+00 |
|  |  | *M. rubriventris* | 16.59130 | 2.867e-07 | 5.786e+07 |
|  |  | *P. sauvagii* | 0.34810 | 9.289e-01 | 3.747e-01 |
|  |  | *R. arenarum* | 0.34615 | 7.900e-01 | 4.382e-01 |
|  |  | Treated | -0.02983 | 5.263e-01 | -5.667e-02 |
|  |  |  |  |  |  |
|  | Intercepts | 0\|1 | -1.2923 | 0.6872 | -1.8806 |
|  |  | 1\|2 | -0.4552 | 0.6664 | -0.6830 |
|  |  |  |  |  |  |
| 4) mFMm.md.tr | Coefficients | Jumper | -1.45509 | 0.6926 | -2.1009 |
|  |  | Treated | -0.06775 | 0.4984 | -0.1359 |
|  |  |  |  |  |  |
|  | Intercepts | 0\|1 | -2.2803 | 0.7142 | -3.1928 |
|  |  | 1\|2 | -1.5465 | 0.6886 | -2.2459 |
|  |  |  |  |  |  |
| 5) mFMm.st.tr | Coefficients | Juvenile | -1.392e+00 | 0.5112 | -2.724e+00 |
|  |  | Treated | 7.514e-06 | 0.5052 | 1.487e-05 |
|  |  |  |  |  |  |
|  | Intercepts | 0\|1 | -1.7761 | 0.4830 | -3.6773 |
|  |  | 1\|2 | -1.0086 | 0.4455 | -2.2640 |
|  |  |  |  |  |  |
| 6) mFMm.sp.st.tr | Coefficients | *L. mystacinus* | -1.45606 | 7.872e-01 | -1.850e+00 |
|  |  | *M. rubriventris* | 16.18755 | 2.702e-07 | 5.992e+07 |
|  |  | *P. sauvagii* | 0.20450 | 9.552e-01 | 2.141e-01 |
|  |  | *R. arenarum* | 0.20508 | 8.130e-01 | 2.522e-01 |
|  |  | Juvenile | -1.19033 | 5.502e-01 | -2.164e+00 |
|  |  | Treated | 0.01131 | 5.414e-01 | 2.090e-02 |
|  |  |  |  |  |  |
|  | Intercepts | 0\|1 | -2.04630e+00 | 7.99700e-01 | -2.55890e+00 |
|  |  | 1\|2 | -1.14940e+00 | 7.67800e-01 | -1.49700e+00 |
|  |  |  |  |  |  |
| 7) mFMm.md.st.tr | Coefficients | Jumper | -1.23545 | 0.7109 | -1.73779 |
|  |  | Juvenile | -1.25012 | 0.5218 | -2.39576 |
|  |  | Treated | -0.03343 | 0.5144 | -0.06498 |
|  |  |  |  |  |  |
|  | Intercepts | 0\|1 | -2.7278 | 0.7674 | -3.5546 |
|  |  | 1\|2 | -1.9324 | 0.7335 | -2.6345 |
|  |  |  |  |  |  |
| 8) mFMm.int1 | Coefficients | *L. mystacinus* | -1.2447 | 1.004e+00 | -1.239e+00 |
|  |  | *M. rubriventris* | 17.8729 | 1.394e-07 | 1.282e+08 |
|  |  | *P. sauvagii* | 17.5566 | 6.864e-01 | 2.558e+01 |
|  |  | *R. arenarum* | 1.3259 | 1.118e+00 | 1.186e+00 |
|  |  | Treated | 1.3258 | 1.373e+00 | 9.658e-01 |
|  |  | *L. mystacinus:*treated | -0.4496 | 1.618e+00 | -2.778e-01 |
|  |  | *M. rubriventris:*treated | -0.6931 | 1.817e-07 | -3.815e+06 |
|  |  | *P. sauvagii:*treated | -18.8824 | 6.864e-01 | -2.751e+01 |
|  |  | *R. arenarum:*treated | -2.1439 | 1.707e+00 | -1.256e+00 |
|  |  |  |  |  |  |
|  | Intercepts | 0\|1 | -8.19000e-01 | 7.985000e-01 | -1.02570e+00 |
|  |  | 1\|2 | 8.220000e-02 | 7.888000e-01 | 1.042000e-01 |
|  |  |  |  |  |  |
| 9) mFMm.int2 | Coefficients | Juvenile | -1.392e+00 | 0.7291 | -1.910e+00 |
|  |  | Treated | -5.951e-06 | 0.7301 | -8.151e-06 |
|  |  | Juvenile: treated | 3.662e-06 | 1.0113 | 3.621e-06 |
|  |  |  |  |  |  |
|  | Intercepts | 0\|1 | -1.776 | 0.5502 | 0.5176 |
|  |  | 1\|2 | -1.0086 | 0.5176 | -1.9486 |
|  |  |  |  |  |  |
| 10) mFMm.int3 | Coefficients | Jumper | -17.07 | 0.4091 | -41.71 |
|  |  | Treated | -16.30 | 0.4130 | -39.46 |
|  |  | Jumper: treated | 16.64 | 0.4939 | 33.70 |
|  |  |  |  |  |  |
|  | Intercepts | 0\|1 | -17.6981 | 0.4238 | -41.7557 |
|  |  | 1\|2 | -16.9443 | 0.4234 | -40.0218 |
|  |  |  |  |  |  |

Results of binomial logistic regression analysis. mNFm: nuclei of the fibrocartilage; mFTM: tendon fibers; mFMm: menisci fibers; mCHm, hypertrophic chondrocytes; st: stage; sp: specie; tr, treatment; md: locomotor mode; int1: intercept sp*tr; int2: intercept st*st.

| **Hypertrophic chondrocytes models** |  | Estimate | Std.error | Z value | Pr(>\|z\|) |
| --- | --- | --- | --- | --- | --- |
| 1) mCH | Intercept | -1.8871 | 0.4799 | -3.932 | 8.42e-05 *** |
|  |  |  |  |  |  |
| 2) mCH.tr | Intercept | -19.57 | 2534.75 | -0.008 | 0.994 |
|  | treated | 18.47 | 2534.75 | -0.008 | 0.994 |
|  |  |  |  |  |  |
| 3) mCH.sp.tr | Intercept | -4.183e+01 | 2.127e+01 | -0.004 | 0.997 |
|  | *L.mystacinus* | 2.194e+01 | 1.018e+04 | 0.002 | 0.998 |
|  | *P. sauvagii* | 1.986e+01 | 1.018e+04 | 0.002 | 0.998 |
|  | *R. arenarum* | -7.516e-02 | 1.187e+04 | 0.000 | 1.000 |
|  | Treated | 2.127e+01 | 5.514e+03 | 0.004 | 0.997 |
|  |  |  |  |  |  |
| 4) mCH.st.tr | Intercept | -20.0962 | 2517.7762 | -0.008 | 0.994 |
|  | Juvenilee | 0.6931 | 1.2450 | 0.557 | 0.578 |
|  | treated | 18.4868 | 2517.7760 | 0.007 | 0.994 |
|  |  |  |  |  |  |
| 5) mCH.sp.st.tr | Intercept | -3.752 | 40909.067 | 0.000 | 1.000 |
|  | *L.mystacinus* | 23.951 | 27813.629 | 0.001 | 0.999 |
|  | *P. sauvagii* | 1.401 | 32549.487 | 0.000 | 1.000 |
|  | *R. arenarum* | -41.208 | 40951.226 | -0.001 | 0.999 |
|  | Juvenile | -41.978 | 26240.812 | -0.002 | 0.999 |
|  | Treated | 23.165 | 14538.694 | 0.002 | 0.999 |
|  |  |  |  |  |  |
| 6) mCH.int1 | Intercept | -2.157e+01 | 1.688e+04 | -0.001 | 0.999 |
|  | *L.mystacinus* | -1.393e-10 | 2.135e+04 | 0.000 | 1.000 |
|  | *P. sauvagii* | -1.382e-10 | 2.669e+04 | 0.000 | 1.000 |
|  | *R. arenarum* | -1.397e-10 | 1.979e+04 | 0.000 | 1.000 |
|  | Treated | -1.390e-10 | 2.387e+04 | 0.000 | 1.000 |
|  | *L. mystacinus:*treated | 2.295e+01 | 2.721e+04 | 0.001 | 0.999 |
|  | *P. sauvagii:*treated | 2.087e+01 | 3.157e+04 | 0.001 | 0.999 |
|  | *R. arenarum:*treated | 1.407e-10 | 2.778e+04 | 0.000 | 1.000 |
|  |  |  |  |  |  |
| 7) mCH.int2 | Intercept | 1.709e+10 | 6.517e+14 | 0.000 | 1.000 |
|  | *L.mystacinus* | 2.008e+01 | 7.929e+03 | 0.003 | 0.998 |
|  | *P. sauvagii* | -1.709e+10 | 6.517e+14 | 0.000 | 1.000 |
|  | *R. arenarum* | -1.709e+10 | 6.517e+14 | 0.000 | 1.000 |
|  | juvenile | -1.709e+10 | 6.517e+14 | 0.000 | 1.000 |
|  | *L. mystacinus:juvenile* | NA | NA | NA | NA |
|  | *P. sauvagii:*juvenile | 1.709e+10 | 6.517e+14 | 0.000 | 1.000 |
|  | *R. arenarum:*juvenile | 1.709e+10 | 6.517e+14 | 0.000 | 1.000 |
